# Physical Therapy-Based Realignment Strategies for Knee Osteoarthritis: A Systematic Review

**DOI:** 10.7759/cureus.110125

**Published:** 2026-06-02

**Authors:** Omkar A Somade, Sandeep Shinde, Vandana M Thorat, Akshaya V Joshi, Mebin S Thomas

**Affiliations:** 1 Department of Musculoskeletal Sciences, Krishna College of Physiotherapy, Krishna Vishwa Vidyapeeth (Deemed To Be University), Karad, IND; 2 Department of Pharmacology and Therapeutics, Krishna Institute of Medical Sciences, Krishna Vishwa Vidyapeeth (Deemed To Be University), Karad, IND

**Keywords:** knee osteoarthritis, lumbopelvic stability, manual therapy, physical therapy, realignment strategies

## Abstract

Knee osteoarthritis (KOA) is a degenerative disorder involving articular cartilage degeneration, causing pain and functional impairment. The condition is most prevalent among individuals in their fourth and fifth decades of life. Biomechanically, musculoskeletal weakness can cause pelvic drop, increasing stress on the knee's medial compartment. While traditional therapy focused on symptomatic relief, current research emphasizes the entire kinetic chain alignment, particularly lumbopelvic stability, lower limb muscle performance to counter joint loading, and abnormal joint reaction forces. The primary objective of this systematic review is to evaluate the current evidence regarding physical therapy-based realignment strategies for individuals diagnosed with KOA.

Adhering to Preferred Reporting Items for Systematic Reviews and Meta-Analyses (PRISMA) guidelines, a systematic search was conducted across databases, including PubMed, Medical Literature Analysis and Retrieval System Online (MEDLINE), and Google Scholar for studies published between 2016 and 2026. The review included 18 randomized controlled trials (RCTs) focusing on conditioning exercises, manual therapy, and realignment strategies. Methodological quality was assessed using the Cochrane Collaboration’s Risk of Bias 2 (RoB 2) tool. This systematic review of 18 RCTs demonstrates that physical therapy-based realignment strategies significantly alleviate pain and improve functional outcomes in patients with KOA. A core finding is the superior effectiveness of a multifaceted kinetic-chain approach over isolated knee protocols. Specifically, proximal strengthening of the hip and core was shown to be equal or superior to isolated knee exercises in enhancing muscle strength and quality of life. Trunk stabilization exercises (TSE), when integrated with knee strengthening, proved more effective at reducing pain and disability than isolated protocols by improving lumbopelvic stability and preventing compensatory biomechanical alterations. Additionally, manual therapy served as a critical adjunct to accelerate early rehabilitation and sustain clinical benefits. Methodological quality assessment using the RoB 2 tool revealed that while the majority of trials demonstrated a low risk of bias in randomization and reporting, the overall evidence profile carries 'Some Concerns' to 'High Risk'. This is primarily attributed to unavoidable challenges in blinding during physical therapy interventions and outcome measurements, which affected 72% of the included studies.

The systematic review concludes that physical therapy interventions utilizing realignment strategies are highly effective for the conservative management of KOA. By transitioning from traditional localized exercises to a multifaceted kinetic-chain approach incorporating proximal hip and core stabilization, clinicians can optimize biomechanical load distribution and neutralize the abnormal joint reaction forces that accelerate cartilage wear. Furthermore, research suggests that even progressive high-impact realignment strategies are safe for individuals with mild KOA, as they enhance bone strength without compromising knee cartilage integrity. Ultimately, these conservative strategies aim to modify the disease's trajectory and restore functional independence, improving the patient's quality of life to such an extent that surgical intervention may be successfully delayed or avoided.

## Introduction and background

Knee osteoarthritis (KOA) is a degenerative joint disorder characterized by progressive wear and tear and loss of articular cartilage. It is a chronic, painful condition that commonly affects the knees, hips, hands, and spine [1]. It most commonly begins in individuals in their fourth and fifth decades of life, with prevalence increasing with age [1]. The severity of symptoms varies among individuals and typically progresses gradually [1]. In advanced stages, KOA can significantly reduce functional capacity and may even lead to work absence [1]. Globally, KOA is estimated to affect approximately 3.8% of the population, with pain being the most common clinical feature [1,2].

Among people with KOA, the disease is most frequently confined to a single compartment at 50%, followed by bicompartmental at 33%, and tricompartmental at just 17%. When looking at specific localized patterns, isolated medial tibiofemoral osteoarthritis (27%), combined medial tibiofemoral and patellofemoral osteoarthritis (23%), and isolated patellofemoral osteoarthritis (18%) are all individually more common than full tricompartmental disease, while patterns involving the lateral tibiofemoral compartment are much rarer, totaling only 15% [3]. This distribution is largely driven by joint mechanics, as the highly prevalent medial compartment osteoarthritis is frequently linked to a varus (bow-legged) knee alignment that increases weight-bearing stress on the inner knee. Conversely, the less common lateral compartment osteoarthritis is typically tied to a valgus (knock-kneed) alignment alongside distinct anatomical and biomechanical risk factors [4].

In KOA, weakness of the surrounding musculature can lead to chronic malalignment, such as pelvic drop (drop of the unilateral pelvis due to weakness in the contralateral gluteus medius), which increases stress on the medial compartment of the knee [2]. Therefore, lumbo-pelvic stability plays a crucial role in maintaining proper load distribution across the knee joint [2]. Evidence suggests that rehabilitation programs targeting proximal muscle groups can reduce pain and improve function, particularly in the short term, with moderate evidence supporting medium- and long-term benefits [5]. Additionally, greater hip abductor strength during gait helps protect the ipsilateral knee from medial compartment KOA, while activation of core muscles such as the transversus abdominis and multifidus enhances trunk stability and supports the lumbopelvic-hip complex during weight-bearing activities [1,5].

People with medial compartment osteoarthritis are also reported to have more pronated feet, while those with lateral compartment osteoarthritis have more supinated feet [2]. A pronated foot posture marked by calcaneal eversion and a flattened medial longitudinal arch forces the tibia into excessive internal rotation, disrupting normal patellofemoral tracking and shifting mechanical stress across the joint space. Conversely, a highly supinated, rigid foot posture limits the lower limb's natural shock-absorption capacity during the stance phase of gait, which shifts the ground reaction force medially, increases the external knee adduction moment, and exacerbates varus (bow-legged) knee malalignment to accelerate medial compartment cartilage degradation [4-6].

The Kellgren-Lawrence scale is a clinical classification system used to categorize the severity of osteoarthritis through specific radiographic features [7]. It ranges from Grade I, characterized by the initial appearance of osteophytes on the joint margins or tibial spines, to more advanced stages involving the formation of periarticular ossicles and the narrowing of joint cartilage [7]. As the condition progresses to Grades III and IV, patients typically exhibit subchondral bone sclerosis, the development of small pseudocystic areas with hardened walls, and visible alterations in the shape of the bone ends [7]. This grading system is essential for clinical research and diagnosis, as it allows practitioners to determine study eligibility and tailor intervention strategies based on the structural extent of the disease [7]. As this structural deformity advances, it expands beyond the cartilage to involve the surrounding bone, triggering radiographic and pathological features such as the formation of marginal osteophytes, significant joint space narrowing, subchondral bone sclerosis, the development of hardened pseudocystic walls, and visible alterations in the contours of the bone ends [8].

Clinical guidelines for KOA prioritize conservative management, with multifaceted conditioning exercises encompassing strengthening, flexibility, endurance, and neuromuscular training serving as the cornerstone for symptom reduction and functional maintenance [9]. While rehabilitation has traditionally centered on the quadriceps, inconsistent evidence regarding its impact on structural disease progression has shifted clinical focus toward the proximal musculature, particularly the lumbo-pelvic stabilizing muscles and hip abductors such as the gluteus maximus, gluteus minimus, gluteus medius, and tensor fasciae latae [1,2]. These muscles are critical for maintaining pelvic stability and proper lower limb alignment, as the pelvis acts as the central regulatory link in the lower extremity kinetic chain. During the single-leg stance phase of dynamic activities like walking or stair climbing, a level pelvis is normally sustained by the isometric and eccentric stabilization of the weight-bearing hip abductors to counteract the gravitational forces acting on the trunk. When this proximal musculature is weak, it fails to maintain this baseline, resulting in a contralateral pelvic drop (or pelvic tilt) that triggers a destructive pathomechanical cascade down the entire kinetic chain. This weakness forces the femur into excessive adduction and internal rotation, disrupting normal patellofemoral tracking and shifting the dynamic ground reaction force vector medially relative to the knee joint center. This medial displacement significantly elevates abnormal joint reaction forces and alters the dynamic external knee adduction moment, concentrating high compressive loads directly onto the medial tibiofemoral compartment, where cartilage wear is most heavily accelerated. Consequently, by targeting these proximal hip abductors alongside traditional quadriceps and hamstrings training, exercise therapy enhances overall joint stability and mechanical load distribution, offering a safe, comprehensive, and cost-effective strategy to optimize long-term functional outcomes for individuals managing KOA [3].

Evidence from systematic reviews and clinical trials indicates that realignment strategies significantly reduce pain, improve physical function, and enhance quality of life in individuals with KOA [9]. Muscle-strengthening and aerobic conditioning are the most commonly prescribed interventions, with over 63.7% of systematic reviews reporting positive outcomes across pain, function, and mobility parameters [9]. Furthermore, realignment strategies have been shown to produce both short-term and long-term improvements in physical performance, supporting their role as a cornerstone in rehabilitation programs [1-5]. While short-term improvements in pain and function have been observed with hip strengthening interventions, the long-term effects on structural changes, function, and disability outcomes are not well established [5]. Considering frameworks such as the disablement model and the International Classification of Functioning (ICF), both functional limitations and disability should be evaluated to fully understand patient outcomes [2-5].

Literature on KOA reveals significant gaps, particularly regarding the long-term efficacy of conditioning exercises on structural disease progression and permanent disability reduction [9]. Current research is marked by high heterogeneity in intervention protocols, treatment durations, and outcome measures, which limits the ability to establish a definitive "gold standard" for exercise combinations [9]. Methodological challenges, such as moderate risks of bias in outcome measurements and deviations from intended interventions, further weaken existing evidence [9]. Consequently, it remains unclear if short-term symptomatic relief translates into delayed surgical necessity or sustained biomechanical correction.

The primary aim of this systematic review is to evaluate the current evidence regarding physical therapy-based realignment strategies for individuals diagnosed with KOA. The study specifically focuses on how these interventions impact critical clinical factors, including the reduction of pain intensity, the improvement of muscle strength, and the restoration of joint range of motion. Furthermore, the objectives include investigating the role of the entire kinetic chain encompassing the spine, pelvis, and lower extremities in influencing joint loading and biomechanics. By synthesizing data from various randomized controlled trials (RCTs), this review seeks to determine the most effective combinations of exercise and realignment strategies to enhance functional ability, mobility, and the overall quality of life for patients managing this degenerative condition.

## Review

Methodology

Study Design

To maintain high levels of clarity and scientific integrity, this systematic review followed the Preferred Reporting Items for Systematic Reviews and Meta-Analyses (PRISMA) framework during the data collection and reporting phases [10].

Search Strategy

A comprehensive and systematic search was conducted across core electronic databases, including PubMed, Medical Literature Analysis and Retrieval System Online (MEDLINE), and Physiotherapy Evidence Database (PEDro). Additionally, Google Scholar and ResearchGate were utilized strictly as secondary, supplementary sources to identify grey literature and ensure no landmark articles were omitted. For the Google Scholar search, screening was limited to the first 100 hits sorted by relevance, after which no further unique or relevant records were identified. Appropriate keywords and Boolean operators (AND, OR) were used to refine the search. The keywords included “knee osteoarthritis,” “medial compartment osteoarthritis,” “realignment strategies,” “conditioning exercises,” “exercise therapy,” “muscle strengthening,” “neuromuscular training,” “physical therapy,” “rehabilitation,” and “functional outcomes.” The literature search was restricted to English-language articles published from January 2016 to April 2026.

Eligibility Criteria

Studies were selected for final inclusion based on strict methodological and biomechanical homogeneity. To ensure exact reproducibility, articles were explicitly chosen only if they investigated physical therapy interventions targeting the wider lower-limb kinetic chain (such as proximal hip stabilization, core strengthening, or gait retraining) to manage joint reaction forces in patients with Kellgren-Lawrence Grades 1-3 knee osteoarthritis. Studies investigating isolated knee protocols without dynamic alignment components, surgical interventions, or purely pharmacological treatments were systematically excluded. The eligibility criteria are listed in Table 1.

**Table 1:** Eligibility criteria

Criteria	Inclusion Criteria	Exclusion Criteria
Population	Patient diagnosed with knee osteoarthritis (KOA)	Patients with other joint disorders or inflammatory conditions (e.g., rheumatoid arthritis), neurological deficits impairing balance or gait (e.g., stroke, Parkinson's), or uncontrolled systemic comorbidities (e.g., severe cardiovascular disease, advanced diabetes, sensory neuropathy)
Age Group	Adults 18-70 years	Pediatric population (<18 years)
Condition	Clinically and/or radiologically diagnosed knee osteoarthritis (Kellgren and Lawrence scale Grades 1-3)	Post-surgical cases or traumatic knee injuries (Kellgren and Lawrence scale Grades 4 and 5)
Intervention	Conditioning exercises, exercise therapy, strengthening programs, neuromuscular training, physiotherapy interventions, and realignment strategies	Pharmacological-only interventions, surgical interventions without rehabilitation
Comparison	Conventional therapy, other exercise programs, or a control group	Studies without comparison groups (if not relevant)
Outcomes	Pain, stiffness, range of motion, functional outcomes, and muscle strength	Studies not reporting relevant clinical outcomes
Study Design	Randomized controlled trials (RCTs)	Case reports, case series, editorials, reviews, pre/post single-arm studies, and non-randomized two-arm studies
Language	Articles published in English	Non-English publications
Publication Type	Full-text, peer-reviewed articles	Abstracts only, unpublished data

Quality Assessment

Methodological quality and risk of bias were evaluated using study-specific validated tools to address the heterogeneity of the research designs. Specifically, the methodological quality and internal validity of the included RCTs were assessed using the Cochrane Collaboration’s Risk of Bias 2 (RoB 2) tool, which systematically evaluates critical study domains including the randomization process, deviations from intended interventions, missing outcome data, measurement of the outcome, and selection of the reported result [11].

Result

**Figure 1:**
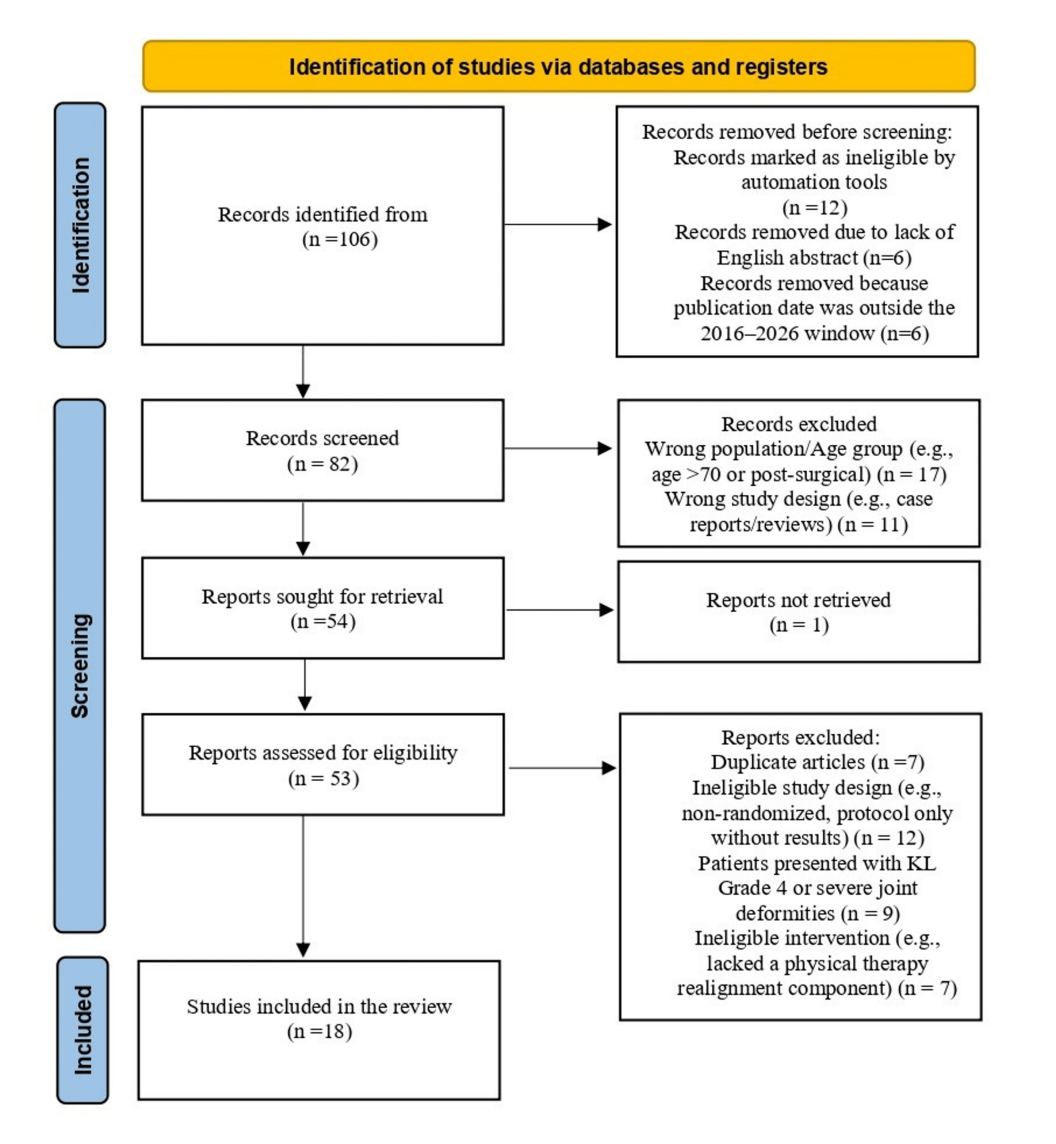
A PRISMA flowchart outlining the study selection process PRISMA: Preferred Reporting Items for Systematic Reviews and Meta-Analysis; KL: Kellgren-Lawrence Scale

A total of 106 records were initially identified through database searching. After comprehensive title, abstract, and full-text screening based on the predefined kinetic-chain eligibility criteria, 18 specific RCTs met all requirements and were selected for final synthesis. Figure 1 shows the PRISMA flow chart [10].

The comprehensive characteristics and selection rationale for the 18 articles are systematically compiled in Table 2.

**Table 2:** Overview of the 18 articles selected for this study 1RM: One-Repetition Maximum; 6-MWT: 6-Minute Walk Test; AROM: Active Knee Joint Range of Motion; dGEMRIC: Delayed Gadolinium-Enhanced MRI of Cartilage; DXA: Dual-Energy X-ray Absorptiometry; EQ-5D-5L: EuroQol 5-Dimension 5-Level Health Questionnaire; FTSST: Five Times Sit-to-Stand Test; GRC: Global Rating of Change Scale; IT: Isometric Training; KOA: Knee Osteoarthritis; KOOS: Knee Injury and Osteoarthritis Outcome Score; LBPP: Lower Body Positive Pressure; LTPA: Leisure-Time Physical Activity; MET: Metabolic Equivalent; MRI: Magnetic Resonance Imaging; MWM: Mulligan's Mobilization With Movement; MWT: Minute Walk Test; NPRS: Numeric Pain Rating Scale; OMERACT-OARSI: Outcome Measures in Rheumatology-Osteoarthritis Research Society International Initiative; RCT: Randomized Controlled Trial; SCT: Stair Climb Test; SF-36: Short Form-36; TSE: Trunk Stabilization Exercises; TUG: Timed Up and Go Test; VAS: Visual Analogue Scale; WOMAC: Western Ontario and McMaster Universities Osteoarthritis Index

Author	Year	Total Participants	Outcome Measures	Kellgren-Lawrence Grade	Intervention	Treatment Frequency	Conclusion
Hasan et al. [12]	2026.	50 (mean age: 54.2 ± 6.8 years, % male: 44%, country: Saudi Arabia)	Knee pain was evaluated using the VAS, while isometric quadriceps strength was quantified via strain-gauge dynamometry. Additionally, patient-reported functional limitations and disability were assessed using the WOMAC.	Grade 2 and Grade 3	MWM combined with IT, compared against the IT alone control group.	6 weeks	The combination of MWM and IT yielded superior and more rapid improvements in clinical outcomes compared to isolated IT. These findings suggest that early-phase MWM application helps mitigate pain during exercise, thereby improving short-term rehabilitative results for individuals with KOA.
Jadhav et al. [13]	2025	34 (% male: 38.2%, Country: India)	Patient outcomes were captured through the KOOS, while functional lower-limb power was assessed using the 30-second chair stand test. Additionally, the Modified Oxford manual muscle grading system was employed to evaluate the strength of the knee extensors and flexors.	Grades 1, 2, and 3	Group A (Experimental): Supervised core stability exercises combined with conventional occupational therapy. Group B (Control/Comparison): Hip strengthening exercises combined with conventional occupational therapy	6 weeks	Both hip and core strengthening exercises are equally effective in improving muscle strength, pain, quality of life, and activities of daily living in patients with KOA. Between-group differences in improvement were found to be statistically insignificant.
Mohamed et al. [14]	2025	60 (country: Saudi Arabia)	WOMAC, Single-leg stance time test for balance, Knee ROM (goniometry), Pain Intensity (NPRS), along with SF-36 for quality of life	Grades 1 to 3	Experimental group: Manual therapy combined with neuromuscular training. Control group: Conventional physical therapy combined with neuromuscular training.	12 months	The application of manual therapy integrated with neuromuscular training yielded significantly superior clinical outcomes compared to conventional physical therapy with neuromuscular training. The experimental group showed a significant reduction in pain at three weeks (mean between-group difference of 1.5; 95% CI: 1.21 to 1.78), which was fully maintained through the six-week follow-up. Patients in the experimental arm also achieved a significantly greater improvement in knee flexion range of motion, functional status, balance, and overall quality of life.
Nazir et al. [15]	2024	60 (% male: 35.6% male, country: Pakistan)	Primary outcome: KOOS. Secondary outcomes: VAS, 6-MWT, and the 11-SCT.	Grades 1 and 2	Group 1 (MWM Protocol): Received six sessions of 6-MWM alongside a foundational regimen of isometric knee strengthening and kinesiotaping. Group 2 (TSE Protocol): Undertook TSE in conjunction with the identical baseline program of isometric knee strengthening and kinesiotaping. Group 3 (Control/Baseline): Performed only the baseline therapy, consisting strictly of isometric knee strengthening and kinesiotaping.	6 weeks	Findings indicated that TSE outperformed both MWM and isolated isometric knee strengthening regarding pain reduction, functional level enhancement, sub-maximal exercise tolerance, and stair-climbing speed. Based on these outcomes, the researchers suggest that practitioners integrate TSE as an effective therapeutic intervention for managing KOA.
Øiestad et al. [16]	2023	168 (mean age: 55.6 ± 8.1 years, % male: 41.7%, country: Norway)	Primary outcome: KOOS quality of life score at one year. Secondary outcome: KOOS subscales (pain, symptoms, activities of daily living, sport/recreation, quality of life at 4 months), knee pain previous week (numeric rating scale), health-related quality of life (EQ-5D-5L index), self-efficacy for pain and function (Arthritis Self-Efficacy Scale), GRC, isokinetic knee extension and flexion strength, and cycle-specific maximal oxygen uptake (VO₂max) at four months and one year.	Grades 2 and 3	12 weeks of strength exercise, aerobic exercise (stationary cycling), or usual care	12 weeks	At the one-year mark, neither exercise regimen yielded a statistically significant impact on KOOS quality of life scores relative to usual care among patients with symptomatic and radiographic KOA. This absence of a therapeutic effect might be attributed to an underpowered sample size. Nevertheless, at the four-month assessment, both the aerobic and strength training cohorts exhibited superior quadriceps muscle strength and higher VO_2_ max levels than the usual care group.
Somaiya et al. [17]	2023	80 (country: India)	Primary outcome: VAS for pain, Hand-held dynamometer for muscle strength, and WOMAC for functional ability. Secondary outcome: 2-MWT	Grade 1 and Grade 2	Group A: Low-intensity plyometric training program along with traditional physiotherapy treatment. Group B: Eccentric training program along with traditional physiotherapy treatment	6 weeks	As this document is a study protocol and patient recruitment has not yet begun, there are no final study conclusions available. The authors hypothesize that through this study, they will be able to create an effective plyometric exercise program to improve physical well-being and performance in individuals with KOA.
Xue et al. [18]	2023	66 (country: China)	Primary outcome: WOMAC scale score for joint function. Secondary outcome: Dynamic and static equilibrium function (measured via Pro-Kin254P balance test system), VAS score for pain, FTSST, and SF-36 for quality of life.	Grade 2 and 3	Treatment group: Electroacupuncture (targeting specific acupoints like Dubi, Neixiyan, Xuehai, Liangqiu, Yanglingquan, and Zusanli) combined with exercise therapy. Control group: Exercise therapy only (focusing on muscle strength and knee mobility training).	4 weeks	As this is a study protocol, definitive clinical conclusions are not yet available. The authors hypothesize that the combination of electroacupuncture and exercise therapy will provide superior rehabilitation benefits in reducing inflammation, alleviating pain, and improving limb function compared to exercise therapy alone.
Rizvi et al. [19]	2023	60 (mean age: 52.41 ± 5.61 years, % male: 0% male, country: Saudi Arabia)	WOMAC, World Health Organization Quality of Life Overall, Heart Rate Variability.	Grade 2	Group 1: Knee strengthening exercises alone (knee reinforcement). Group 2: Integrated polyvagal theory-based exercises combined with knee-strengthening exercises.	6 weeks	The combination of polyvagal theory-based exercises and knee strengthening training yielded significantly superior outcomes compared to knee strengthening exercises alone in women with grade II KOA. Group 2 achieved greater reductions in joint pain, stiffness, and functional limitations, improved psychological and social quality of life domains, and demonstrated optimized autonomic regulation. The findings suggest that incorporating polyvagal exercises serves as a highly beneficial, non-invasive adjunctive strategy for managing KOA.
Zhao et al. [20]	2023	330 (Country: China)	Primary outcome: Proportion of participants achieving a minimal clinically important improvement on the WOMAC pain dimension at 8 and 26 weeks. Secondary outcome: WOMAC total, stiffness, and function scores; Patient Global Assessment; OMERACT-OARSI responder index; SF-12 Health Survey; Arthritis Self-Efficacy Scale; and EQ-5D-5L.	Grade 2 or 3	Acupuncture: Semi-standardized treatment involving six essential acupoints and three adjunct acupoints based on pain location. Tuina: A standardized six-step manual therapy involving relaxation, acupressure, plucking, and joint movement. Home-based exercise (Control): A stepped exercise program delivered via a mobile app ("Knee for long").	4 weeks	As this is a study protocol, a final conclusion on effectiveness is not yet available; however, the study aims to provide high-quality evidence for the selection of KOA treatments and enhance the clinical application of acupuncture and tuina.
Nigam et al. [21]	2021	35 (mean age: 55.12 ± 5.60 years, % male: 35% male, country: India)	Primary outcome: WOMAC. Secondary outcomes: Pain intensity (24-hour and sit-to-stand) measured on a 10-cm VAS, TUG test, 12-step stair test, knee ROM, and patient satisfaction (11-point Numerical Rating Scale)	Grades 1, 2, and 3	Experimental group: Participants received MWM integrated into a standard care regimen consisting of therapeutic exercise and moist heat applications. Control group: Participants underwent standard care exclusively, which comprised identical therapeutic exercise and moist heat protocols.	2 weeks	The addition of MWM to usual care provides clinically significant long-term improvements in disability, pain, functional activities, and patient satisfaction for patients with symptomatic KOA, with benefits sustained at least six months later
Chen et al. [22]	2021	18 (mean age: 62.13 ± 8.24 years, % male: 20% male, country: China)	Primary outcome: The principal investigation focused on spatiotemporal and kinematic gait parameters quantified through three-dimensional gait analysis. Specific metrics included average walking velocity, cadence, stride length, step width, the duration of stance and swing phases, and sagittal plane knee flexion-extension angles. Secondary outcome: Supplemental measures encompassed a suite of clinical assessments: the WOMAC to evaluate physical function, a VAS for subjective pain tracking, active knee range-of-motion (AROM) via goniometry, and the modified Barthel index to determine independence in activities of daily living.	Grade 2 and 3	LBPP group: LBPP treadmill walking training program. Control group: Conventional walking on the indoor ground at a self-selected speed. (Both groups also maintained their daily conventional physiotherapy and manual therapy)	2 weeks	The LBPP training group experienced a significantly greater improvement in gait parameters such as walking speed, stride length, and knee range of motion compared to the conventional walking group. While both groups saw improvements in pain and clinical assessments, the LBPP treadmill exercise training is considered an effective approach for alleviating pain symptoms and enhancing lower extremity locomotion in patients with mild to moderate KOA.
Chao et al. [23]	2021	166 (mean age: 56.0 ± 10.5 years, % male: 41.6% male, country: China)	Patient clinical outcomes and functional status were evaluated utilizing the Lysholm Knee Score, the WOMAC, the SF-36 Health Survey, and goniometric measurements of knee ROM.	Grade 1-3	Participants were allocated into two distinct cohorts: an experimental group that underwent a structured, systematic exercise rehabilitation program, and an active control group receiving standard pharmacological management. The control cohort consisted of patients prescribed either naproxen (n = 28), diclofenac (n = 27), or celecoxib (n = 19).	12 weeks	Over a 12-week follow-up period, a structured exercise rehabilitation program demonstrated superior efficacy in mitigating symptoms and enhancing quality of life for patients with KOA compared to conventional treatment with nonsteroidal anti-inflammatory drugs and selective COX-2 inhibitors.
Reza et al. [24]	2021	32 (mean age: 53.8 ± 6.1 years, % male: 40% male, country: Saudi Arabia)	NPRS, WOMAC	Grade 1 and 3	Participants were randomly allocated into two groups: Group A: Received a supervised exercise protocol (consisting of both strengthening and stretching exercises). Participants assigned to Group B underwent a combined therapeutic regimen consisting of targeted manual therapies, specifically myofascial mobilization and joint manipulation techniques integrated alongside the supervised exercise protocol.	4 weeks	The integration of targeted manual therapies with a supervised exercise regimen demonstrated significantly greater efficacy in mitigating pain intensity and ameliorating functional disability in patients with KOA than the exercise protocol utilized in isolation.
Messier et al. [25]	2021	377 (mean age: 65.0 ± 7.8 years, % male: 30.2% male, racial and ethnic breakdown: White: 79.8% (301 participants), Black or African American: 17.5% (66 participants), Hispanic: 1.6% (6 participants), Other (including Asian or Native American): 1.1% [4 participants), country: United States)	Primary outcomes: Knee pain measured by WOMAC (0 best to 20 worst) and knee joint compressive force (maximal tibiofemoral contact force) at 18 months. Secondary outcomes: These included physical function and other clinical symptoms	Grade 2 or 3	High-intensity strength training: resistance training using 75% to 90% of the 1RM, Low-intensity strength training: resistance training using 30% to 40% of 1RM, Attention control group: a group that received healthy living workshops and was used for comparison.	18 months	At the 18-month follow-up, high-intensity resistance training failed to yield a statistically significant reduction in either knee pain or tibiofemoral compressive forces among adults with KOA when compared to low-intensity exercise or an attention control group. Consequently, these findings do not demonstrate any therapeutic superiority of high-intensity protocols over lower-intensity alternatives or control conditions for these specific clinical outcomes
Alghadir et al. [26]	2019	68 (mean age: 53.7 ± 6.9 years, % male: 44.3% male, country: Saudi Arabia)	Primary outcomes: The primary outcome measures consisted of average pain levels and knee functional capacity, which were quantified using the NPRS and WOMAC, respectively. Secondary outcomes were quadriceps muscle strength and TUG test scores.	Grades 1 to 3	Retro Walking group: Supervised retro walking training in addition to routine physiotherapy. Forward Walking group: Supervised forward walking training in addition to routine physiotherapy. Control group: Routine physiotherapy program consisting of a combination of closed and open kinematic chain exercises.	6 weeks	Following a six-week intervention, a retro walking program yielded significantly greater reductions in pain and functional disability alongside superior improvements in quadriceps muscle strength and objective physical performance metrics compared to both forward walking and standard control conditions.
Nazari et al. [27]	2018	93 (mean age: 58.11 ± 7.91 years, % male: 24.4% male, country: Iran)	Pain intensity measured by the VAS, active knee flexion ROM, TUG, 6-MWT, and the WOMAC total and subscales	Grade 2 and 3	High-intensity laser therapy group: Received pulsed Nd: YAG laser therapy alongside a standardized exercise protocol. Conventional physical therapy group: Received transcutaneous electric nerve stimulation and continuous ultrasound in addition to the standardized exercise protocol. Exercise therapy group: Received the standardized exercise protocol alone as a home-based program	4 weeks	High-intensity laser therapy combined with exercise therapy proved more effective than both conventional physical therapy with exercise and exercise therapy alone in improving the pain and functional outcomes for patients with KOA.
Kuru et al. [28]	2017	78 (mean age: 56.5 ± 7.9 years, % male: 14.6% male, country: Turkey)	Pain was assessed using a VAS. Quadriceps and hamstring muscle strengths were measured with a dynamometer. Functional capacity was evaluated via the 6-MWT. Balance was assessed using an electronic balance board. Non-invasive hemodynamic parameters were monitored using impedance cardiography.	Grade 2 and 3	The exercise intervention comprised low-resistance static and dynamic contractions targeting the primary muscle groups of the lower limbs, integrated with fundamental postural stability drills. Participants were randomized into one of two implementation tracks: an outpatient, clinic-supervised training group or a self-administered, home-based exercise cohort.	6 weeks	A clinic-supervised protocol of low-intensity lower limb exercises under physiotherapist guidance demonstrated superior efficacy to a home-based program in alleviating post-activity pain and enhancing muscular strength in the quadriceps and right hamstrings. Notably, both intervention delivery models were equally successful in mitigating resting pain and extending the 6-minute walk distance.
Multanen et al. [29]	2016	80 (mean age: 57.9 ± 5.0 years, % male: 0% male, country: Finland)	To assess the structural integrity of the femoral neck, DXA was utilized to quantify the subperiosteal width, cross-sectional area, and section modulus (indicative of bending strength). Concurrently, the biochemical profile of the knee cartilage was analyzed via quantitative MRI protocols, specifically employing T_2_ mapping and dGEMRIC. Additionally, cumulative physical activity exposure was captured using accelerometer-based motion sensors.	Grade 1 and 2	A progressive, supervised high-impact aerobic and step-aerobic exercise program. The exercises included jumping and rapid changes of direction to music, with the loading progressively increased every three months by adjusting stepping and obstacle heights.	12 months	Progressive high-impact training significantly increased femoral neck bending strength in postmenopausal women with mild KOA. This improvement in bone strength was achieved without any harmful or detrimental effects on the biochemical composition of the weight-bearing knee cartilage

Risk of Bias Assessment

To ensure the credibility of the synthesized data, a risk of bias evaluation was performed for each selected RCT. The methodological quality and internal validity of the selected literature were scrutinized using the RoB 2 [11]. This validated instrument assesses five core methodological domains: the randomization process, deviations from intended interventions, missing outcome data, measurement of the outcome, and selection of the reported result.

Using the RoB 2 tool, the risk of bias assessment across the 18 included RCTs revealed varied results. Four studies, Xue et al. [18], Nigam et al. [21], Messier et al. [25], and Multanen et al. [29] demonstrated a robust methodological profile, receiving 'Low Risk' ratings across all five domains. Conversely, the study by Mohamed et al. [14] exhibited a high risk of bias across all five domains. The remaining 13 studies were classified as having 'Some Concerns' overall; this was primarily driven by the 'measurement of outcomes' domain, where minor methodological vulnerabilities were consistently identified. Other isolated issues were noted in the implementation and data tracking domains: the trial by Rizvi et al. [19] exhibited distinct risks regarding both deviations from intended interventions and missing outcome data, while the study by Alghadir et al. [26] was similarly appraised as having "Some Concerns" due to incomplete outcome data tracking. Notably, objective review confirmed that all 18 evaluated trials demonstrated strong internal validity regarding the randomization process and the selection of reported results, universally meeting the criteria for a low-risk designation in both categories. Table 3 presents the formal risk of bias assessment across all included RCTs using the RoB 2 framework.

**Table 3:** Risk of bias assessment of randomized control trials using Cochrane Collaboration’s Risk of Bias 2 (RoB 2) tool

Study	Randomization Process	Deviations from Intended Interventions	Missing Outcome Data	Measurement of the Outcome	Selection of the Reported Result	Overall Risk of Bias
Hasan et al. [12]	Low	Low	Low	Some concerns	Low	Some concerns
Jadhav et al. [13]	Low	Low	Low	Some concerns	Low	Some concerns
Mohamed SH et al. [14]	Low	High	Low	High	Low	High
Nazir et al. [15]	Low	Low	Low	Some concerns	Low	Some concerns
Oiestad et al. [16]	Low	Low	Low	Some concerns	Low	Some concerns
Somaiya e al. [17]	Low	Low	Low	Some concerns	Low	Some concerns
Xue et al. [18]	Low	Low	Low	Low	Low	Low
Rizvi et al. [19]	Low	Some concerns	Some concerns	Some concerns	Low	Some concerns
Zhao et al. [20]	Low	Low	Low	Some concerns	Low	Some concerns
Nigam et al. [21]	Low	Low	Low	Low	Low	Low
Chen et al. [22]	Low	Low	Low	Some concerns	Low	Some concerns
Chao et al. [23]	Low	Low	Low	Some concerns	Low	Some concerns
Reza et al. [24]	Low	Low	Low	Some concerns	Low	Some concerns
Messier et al. [25]	Low	Low	Low	Low	Low	Low
Alghadir et al. [ 26]	Low	Low	Some concerns	Some concerns	Low	Some concerns
Nazari et al. [27]	Low	Low	Low	Some concerns	Low	Some concerns
Kuru et al. [28]	Low	Low	Low	Some concerns	Low	Some concerns
Multanen et al. [29]	Low	Low	Low	Low	Low	Low

Discussion 

Eighteen RCTs met the eligibility criteria and were included in this systematic review to comprehensively evaluate the efficacy of physical therapy-based realignment and conditioning strategies for KOA. Collectively, the compiled evidence marks a definitive paradigm shift in conservative KOA rehabilitation, transitioning from historically localized knee interventions toward a multifaceted, kinetic-chain approach [5]. The consolidated results demonstrate that proximal musculature conditioning specifically targeting core and hip abductor stabilization is highly effective, with trunk stabilization yielding equal or superior efficacy to isolated lower limb training for improving both pain and long-term functional capacity [13]. Biomechanically, these findings validate the clinical necessity of lumbopelvic-hip complex stability in neutralizing pathomechanical forces across the lower extremity.

Extensive evidence underlines the critical impact of proximal musculature on regulating biomechanical forces across the knee joint [13]. In alignment with this, Jadhav et al. demonstrated that hip-targeted and core-stabilization training protocols yield comparable efficacy in alleviating pain, augmenting muscular strength, and enhancing overall quality of life in individuals suffering from KOA [13]. However, further investigation by Nazir et al. suggests that trunk stabilization exercises, when combined with isometric knee strengthening and kinesiotaping, demonstrate greater efficacy in reducing pain and enhancing functional capacity compared to either Mulligan's mobilization or isolated knee strengthening [15]. This supports the theory that improving lumbopelvic-hip stability prevents biomechanical alterations like pelvic drop, which otherwise increases stress on the medial compartment of the knee.

Manual therapy serves as a powerful adjunct to exercise protocols [12]. Hasan et al. found that integrating mobilization with movement (MWM) into an isometric training program significantly accelerated and amplified six-week improvements in pain and functional status [12]. These benefits appear to be durable, as Nigam et al. reported that the addition of MWM to usual care yielded clinically significant improvements that were sustained for at least six months [21]. The integration of targeted manual techniques, specifically myofascial mobilization and joint manipulation, with a supervised exercise program yielded significantly greater improvements in mitigating both pain intensity and functional disability compared to the exercise protocol utilized in isolation [21].

Advanced gait retraining and technological modalities offer superior outcomes compared to conventional walking [26]. Research by Alghadir et al. revealed that a six-week regimen of backward walking yielded superior results compared to forward walking or standard physical therapy, specifically demonstrating more pronounced decreases in pain and disability alongside greater gains in quadriceps strength [26]. Similarly, the use of a lower-body positive-pressure treadmill was shown to improve gait parameters such as walking speed and stride length more effectively than indoor walking on solid ground [22].

Manual therapy, specifically MWM, was found to significantly accelerate early rehabilitation outcomes when combined with isometric training [12,21]. Innovative approaches such as retro-walking and LBPP treadmill training outperformed conventional walking in enhancing gait parameters and reducing pain [22,26]. Interestingly, high-intensity strength training did not prove superior to low-intensity training at 18 months, suggesting that load intensity may be less critical than consistent adherence [25].

Clinical Implications

Clinically, these findings suggest that a multifaceted, kinetic-chain approach is essential [5]. Physiotherapists should move beyond local knee strengthening to incorporate trunk stabilization and hip abductor training to optimize mechanical load distribution. Additionally, supervised clinic-based programs appear more effective than home-based exercises for immediate pain relief and strength gains [28]. The inclusion of manual therapies and specialized walking programs (like retro-walking) can further enhance the speed of functional recovery [12,21,26].

Beyond short-term symptom relief, a vital objective of kinetic-chain physical therapy is postponing invasive arthroplasty. While a definitive timeframe cannot be universally generalized due to patient-specific factors such as baseline body mass index, structural pathomechanics, and rehabilitation compliance, longitudinal data suggest that structured conservative management can successfully delay total knee arthroplasty by an average of two to five years in individuals with Kellgren-Lawrence Grade 1-3 degeneration [12]. This therapeutic window preserves functional independence and enhances post-surgical prognosis by optimizing baseline muscular architecture before eventual operative intervention.

Limitations and Future Scope

A primary limitation of the current literature is the "Some Concerns" rating in the measurement of outcomes for 72% of the studies, largely due to the inherent difficulty of blinding both participants and personnel in physical therapy trials. High heterogeneity in treatment protocols also prevents the establishment of a definitive gold standard for exercise combinations. Furthermore, restricting the database search to the past 10 years (2016-2026) introduces a potential risk of omission, as it may overlook landmark or highly significant foundational studies in this area. Similarly, the eligibility criteria exclusively favored RCTs; while this ensures a high level of methodological rigor, it inherently excludes lower-rigor designs that might still offer important, practical contributions to the field.

## Conclusions

The systematic review concludes that physical therapy interventions utilizing realignment strategies are highly effective for the conservative management of KOA. By transitioning from traditional localized exercises to a multifaceted kinetic-chain approach incorporating proximal hip and core stabilization, clinicians can optimize biomechanical load distribution and neutralize the abnormal joint reaction forces that accelerate cartilage wear. Furthermore, preliminary evidence from isolated high-quality trials within this review suggests that specialized, progressive high-impact loading protocols may be safe for select individuals with mild (early-stage) KOA, as they enhance bone strength without compromising knee cartilage integrity. Ultimately, these conservative strategies aim to modify the disease's trajectory and restore functional independence, improving the patient's quality of life to such an extent that the necessity for invasive surgical intervention may be significantly delayed, while ensuring optimal functional preservation throughout the disease's progressive course.
